# A Case of Paratesticular Cellular Angiofibroma Presenting as Inguinoscrotal Hernia

**DOI:** 10.1002/ccr3.70600

**Published:** 2025-07-06

**Authors:** Hamza Arif, Muhammad Shaheer Bin Faheem, Munawer Latif Memon, Khawaja Shaheryar, Syed Abdullah Monawwer, Sumaya Samadi

**Affiliations:** ^1^ Department of Surgery POF Hospital Wah Cantt Pakistan; ^2^ Department of Medicine and Surgery Karachi Institute of Medical Sciences, KIMS Karachi Pakistan; ^3^ Ziauddin Medical University Karachi Pakistan; ^4^ Kabul University of Medical Sciences “Abu Ali Ibn Sina” Kabul Afghanistan

**Keywords:** benign neoplasm, cellular angiofibroma, histopathology, immunohistochemistry, tumor

## Abstract

Cellular Angiofibroma (CAF) is a rare and benign neoplasm with a good prognosis. Diagnosis of CAF can be challenging due to its nonspecific clinical and radiological features, which can often lead to a misdiagnosis, as in our case of a 77‐year‐old male. Histopathology and immunohistochemistry play a crucial role in identifying CAF and differentiating it from malignant tumors. Although not many cases of recurrence have been reported, long‐term follow‐up should still be considered.

## 1 Introduction

Cellular Angiofibroma (CAF) is a rare, benign tumor and densely vascularized fibroblastic neoplasm that develops in the vulvovaginal region in females and the inguinoscrotal region in males. CAF of the vulva was first reported in women in 1998, and a year later, angiofibroblastoma tumor was also reported in the inguinoscrotal region of males [1, 2]. These soft tissue tumors are usually confined to inguinoscrotal regions, but other sites have also been reported. Histologically, spindle cells containing short bundles of wispy collagen and myxoid stroma with small to medium‐sized vessels are its characteristic features, among others. Although the complete pathogenesis of CAF is not known, chromosomal aberrations and hormonal influences are credited for part of it [3].

The most common presentation in patients is a slow‐growing, painless mass with few reports of intermittent genital bleeding and pain [3]. Clinically, para testicular angiofibroma is difficult to suspect due to its association with hernia or hydrocele [4]. Moreover, due to its rare incidence and usual presentation as a slowly enlarging and painless mass in the scrotum, it can easily lead to them being presumed as inguinal hernia in clinical practice [4, 5]. It is also imperative for surgeons to timely diagnose and differentiate scrotal masses in men from other aggressive tumors. Here, we present a case of a 77‐year‐old male with paratesticular CAF who presented as incarcerated inguinal hernia.

## 2 Case History/Examination

A 77‐year‐old male presented to the emergency department with sudden onset, non‐radiating pain in the left inguinoscrotal mass for the past 5 days. The swelling was first noticed 10 years back and gradually increased in size. A review of systems was unremarkable, with no history of fever, vomiting, constipation, or any urinary tract symptoms. No previous workup was done for this swelling.

On examination, an approximately 20 × 13 cm pyriform‐shaped swelling of the left inguinoscrotal region was noted. It was slightly tender to the touch, firm in consistency, and not adherent to the overlying skin. A cough impulse was absent, and the left testis was not palpable due to the massive size of the swelling. Reduction maneuvers were not successful, and he was initially diagnosed with an incarcerated inguinal hernia.

Ultrasonography showed unremarkable bilateral testes with an abdominal wall defect in the left inguinal region. Mesenteric fat was herniating through the defect and reaching up to the ipsilateral scrotal sac. Color Doppler Imaging (CDI) showed minimal internal vascularity, raising suspicion of left‐sided incarcerated inguinoscrotal hernia.

## 3 Differential Diagnosis

The differential diagnosis for this case included incarcerated inguinal hernia, strangulated hernia, and hydrocele. Although CAF was part of the differential diagnosis, initial clinical and radiological presentations did not support it.

## 4 Conclusion and Results

**FIGURE 1:**
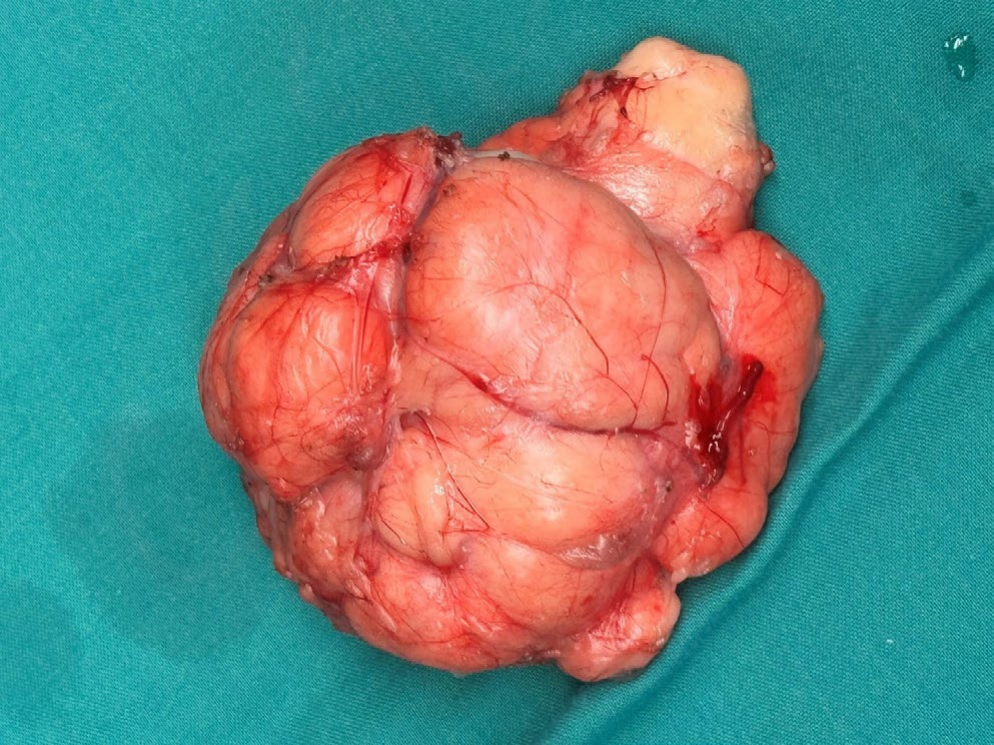
Surgical specimen of Cellular Angiofibroma.

The patient underwent emergency surgery. A skin crease incision was made approximately 4 cm above and parallel to the inguinal ligament, which was later extended inferiorly to gain access to the scrotum. Intraoperative findings revealed an anterior abdominal wall defect of 2 cm in the left inguinal region. A well‐circumscribed, yellowish‐pink mass, measuring 15 × 10 × 6 cm, resembling fat tissue with rich vasculature, was also found in the left hemi‐scrotum, as shown in Figure 1. The mass appeared to arise from para testicular tissue, isolated from the left testis and adherent to but not invading the spermatic cord. Testis‐sparing surgery was performed. The mass was carefully isolated from surrounding structures, excised, and sent for histopathology and immunohistochemistry. The abdominal wall defect was repaired using a 6 × 11 cm Prolene mesh. Tumor markers were also within the normal range, including serum alpha‐fetoprotein (AFP) and human chorionic gonadotrophin (hCG).

Histopathology revealed a well‐circumscribed tissue with a homogenous gray‐white cut surface. Microscopic examination showed medium‐sized blood vessels surrounded by spindle cells arranged in fascicles, along with hyalinized stroma infiltrated by lymphocytes and mast cells. Immunohistochemistry was positive for Epithelial Membrane Antigen (EMA) with very focal CD34; the Ki‐67 proliferation index was less than 1%.

No post‐operative complications were noted, and the patient was discharged 3 days later and kept on regular follow‐ups.

## 5 Discussion

Cellular Angiofibromas (CAF) are rare mesenchymal tumors that often grow slowly. They show peak incidence in the 70s for men and 50s for women. These tumors range from 0.6 to 25 cm and are often smaller in women. Macroscopically, they can be seen as oval, round, or lobulated, well‐circumscribed nodules with soft to rubbery consistency. On the cut surface, they are solid with a yellowish‐brown to grayish‐pink color [4]. According to histopathology reports, CAFs are well‐circumscribed, composed of invariable, short spindle‐shaped cells, often found in a fascicular arrangement or nuclear palisading. Numerous small‐ to medium‐sized thick‐walled blood vessels, along with short, wispy collagen bundles, can also be seen in their fibrous stroma. Perivascular lymphoid aggregates were also noted. Moreover, hyalinization or myxoid changes were also seen, especially in men, along with variable stromal edema. In 50% of the cases, frequent mast cells and small aggregates or individual adipocytes were observed [3, 4]. The differential diagnosis based on histopathology and immunohistochemistry included Solitary Fibrous Tumor (SFT), Angiomyofibroblastoma (AMFB), and Myofibroblastoma, among others; however, these can be differentiated pathologically. SFT has a patternless architecture with spindle cells and a very distinct staghorn‐shaped vasculature, while on immunohistochemistry it is typically CD34 positive, negative or focal EMA, and shows strong nuclear positivity for STAT6, which distinguishes it [6]. Similarly, AMFB is a hypervascular tumor with alternating and myxoid areas that contain bland spindle and epithelioid cells. They show variable positivity for CD34 and can be EMA positive; however, the absence of hypervascularity aids in this differentiation [7] The combination of histological key features, immunohistochemistry, and clinical presentation can aid in the diagnosis of Cellular Angiofibroma. Our patient's macroscopic and histopathological findings were consistent with the characteristic features of CAF, further supporting the diagnosis.

Iwasa et al. showed that immunohistochemistry stains of CFA express progesterone and estrogen receptors in numerous cases and CD34 in around 30 to 60% of the cases. A few cases also noted variable expressions of Smooth Muscle Actin (SMA) and Desmin [4]. Our patient's immunohistochemistry report showed CD34 and positive EMA, consistent with previously reported findings. The Ki‐67 proliferation index was also less than 1%, showing CFA's slow growth rate. A molecular diagnosis is usually not required. However, Arakaki et al. showed that a monoallelic deletion of 13q14 and associated downregulation of *RB1* and *FOXO1*, both encoded in 13q1, has been reported in cases of CAF. This suggests a common genetic and molecular mechanism for tumorigenesis [8].

Ultrasonography with Color Doppler, CT scan, and MRI can be used as imaging modalities to diagnose CAF. However, CAF has varied and non‐specific radiological findings. Some tumors may show areas of increased intensity on T2‐weighted MRI images or changes in density on CT scans owing to their vascularity [9, 10].

In the case of a benign paratesticular mass with negative markers of the tumor, testis‐sparing surgical excision with tumor‐free margins is the treatment of choice. Radical orchiectomy is also preferred in cases where benign and malignant masses cannot be differentiated [11]. In this case, a definitive diagnosis was not available initially, so a urologist was also consulted during the procedure, and the tumor was separable from the testis and spermatic cord. As the mass seemed benign according to clinical and immunohistochemistry findings, it was decided to spare these organs.

It has also been reported that patients who underwent complete tumor excision had good prognoses without any recurrence [3]. Unfortunately, follow‐up data for CAF is limited, but rare cases of recurrences have been reported [2, 12]. Thus, long‐term follow‐up is required. This should also prompt physicians to work up any masses the patients present on time to catch any malignant disease and report new cases with follow‐up to expand the current knowledge pool.

## Author Contributions

**Hamza Arif:** conceptualization, data curation, project administration, validation, visualization, writing – original draft. **Muhammad Shaheer Bin Faheem:** conceptualization, data curation, project administration, resources, supervision, visualization, writing – original draft, writing – review and editing. **Munawer Latif Memon:** resources, validation, writing – original draft, writing – review and editing. **Khawaja Shaheryar:** resources, validation, writing – original draft. **Syed Abdullah Monawwer:** investigation, methodology. **Sumaya Samadi:** investigation, methodology, writing – review and editing.

## Ethics Statement

The authors have nothing to report.

## Consent

Written permission given by patient.

## Conflicts of Interest

The authors declare no conflicts of interest.

## Data Availability

Data sharing is available from corresponding author upon reasonable request.
